# Compliance towards post-exposure prophylaxis of rabies vaccination among animal bite patients at a tertiary care hospital in Khandwa, Madhya Pradesh: A retrospective study

**DOI:** 10.6026/973206300223437

**Published:** 2026-06-30

**Authors:** Soumitra Sethia, Sachin Parmar, Admira Fernandes, Durga Ahujam, Priyaranjan Singh, Shreyash Dwivedi, Souryakant Varandani

**Affiliations:** 1 Department of Community Medicine, Sunderlal Patwa Government Medical College, Mandsaur, Madhya Pradesh, India; 2 Department of Community Medicine, V.K.S. Government Medical College, Neemuch, Madhya Pradesh, India; 3 Department of Community Medicine, NandKumar Singh Chauhan Medical College, Khandwa, Madhya Pradesh, India; 4 Department of Pharmacology, NandKumar Singh Chauhan Medical College, Khandwa, Madhya Pradesh, India

**Keywords:** Rabies, post-exposure prophylaxis, anti-rabies vaccine

## Abstract

Rabies caused by lyssavirus is almost always deadly, with 59,000 deaths occurring in Asia and Africa due to poor access to PEP(Post- exposure prophylaxis). Hence, aretrospective study of 850 animal bite cases (April-September 2023) was conducted at a tertiary care hospital in Khandwa, Madhya Pradesh. Most patients were males (74.9%) and aged 21-30 years (23.9%), with Category II exposure being the most common (84%). Only 44.8% completed the four-dose intradermal ARV regime, with a high dropout rate of 55.2% especially after Dose 3. Thus, we show a major compliance gap, emphasizing the need for improved follow-up systems and targeted health education.

## Background:

Rabies is almost always fatal zoonotic encephalitis caused by lyssaviruses and remains a persistent global public health problem. Although global age-standardized incidence rates declined by 69.4% from 1990 to 2021, significant disparities persist in low socio- demographic index regions, particularly among children and older adults [1]. Rabies burden is strongly associated with sociodemographic development, with higher morbidity and mortality in low-income countries. In 2019, there were an estimated 14,075 cases (95% UI: 6,124-21,618) and 13,743 deaths (95% UI: 6,019-17,938) globally [2]. India accounts for about 36% of global rabies deaths yearly. Dog bites cause 95-97% of all human cases, and an estimated 17.4 million dog bites occur annually in India, leading to around 18,000-20,000 rabies death each year [3]. A geospatial analysis found that 6.6 per 1,000 people in India experience dog bite annually, totally about 9.1 million cases each year, with nearly half of vaccine recipients not completing the full course. India is the leading contributor to rabies mortality, accounting for 65% of all rabies deaths in the South-EastAsian region [4].The WHO sorts animal bites into three groups: Category I (no exposure), Category II (minor exposure that needs vaccination), and Category III (severe exposure that needs vaccination and rabies immunoglobulin or monoclonal antibodies) [5]. These groups are the basis for making PEP (Post-exposure prophylaxis) decisions and the most common clinical presentation of animal bites is Category III exposures, which make up 55.57% of all cases. Category II exposures make up 41.18% of cases, and Category I exposures make up 3.25% [6]. PEP involves immediate wound washing with soap and water for at least 15 minutes, administration of RIG/monoclonal antibodies (Rabies immunoglobulin) for Category III exposures, and starting the WHO-recommended one-week intradermal vaccination schedule. Public education on the PEP process is essential to ensure adherence and prevent misconceptions about rabies risk [7]. A hospital-based study from Jammu found that only 71.7% of animal bite victims completed the full anti-rabies vaccine course, even though PEP is known to work [8]. A systematic review and meta-analysis of 30 Indian studies (n=35,600) indicated a pooled PEP compliance rate of merely 57% (95% CI: 48-66%), with adherence decreasing with successive doses and being markedly higher for Category III bites [9]. PEP dropout was significantly correlated with perception bias and a consistent pattern of low attendance at healthcare and vaccination centers, particularly among individuals who did not regularly pursue care for other illnesses [10]. In Côte d'Ivoire, the main reason for PEP abandonment was lack of money (41.5%). Patients who chose the 5-dose protocol dropped out much more often (P≤0.001) than those who chose shorter schedules [11]. In Vietnam, even though PEP was widely available, the rates of people completing the vaccine regime were low due to factors like the patients' characteristics and provincial rabies burden, this shows that providers need more training and targeted education [12]. Incomplete PEP schedules significantly diminish the probability of attaining protective rabies virus-neutralizing antibody titres and it was seen among 54 breakthrough rabies cases adhering strictly to core PEP protocols, merely 44% had completed the entire vaccine series [13].Systemic challenges in India, such as high RIG costs, anti-rabies clinics being hard to get to, a lack of cold-chain infrastructure, healthcare workers not knowing enough, and low community awareness, continue to make it hard for people to get the best PEP. Strengthening surveillance, making sure that everyone gets the same level of service, and providing health education that is appropriate for the situation are still important steps toward the WHO's goal of having no human deaths from rabies by 2030 [14]. Therefore, it is of interest to evaluate the compliance towards post exposure prophylaxis of rabies vaccination.

## Materials and Methods:

## Study design and setting:

A retrospective, observational, record-based study was performed at the Anti-Rabies Clinic (ARC) of the Tertiary Care Hospital in Khandwa, Madhya Pradesh. The ARC is the main place for people in the Khandwa district who have been bitten by an animal to get care. It follows the National Rabies Control Programme (NRCP) guidelines for post-exposure prophylaxis (PEP). Length of Study. The research was carried out over six months, spanning from April 2023 to September 2023.Study Group the study population consisted of all animal bite patients who visited the ARC during the study period and received their initial dose (Day 0) of anti-rabies vaccination. The study included 850 patients who came to the ARC after being bitten by an animal.

## Inclusion criteria: 

[1] All animal bite victims who visited the ARC for the first time (zero dose) between April 2023 and September 2023

[2] Patients of all ages and both genders

## Criteria for exclusion:

[1] Cases where the ARC records don't have enough, complete, or readable information

[2] Patients who had already started PEP at another place before coming to the ARC Schedule for vaccinations

All eligible patients received PEP through Intradermal (ID) Rabies Vaccination utilizing the Updated Thai Red Cross (UTC) regimen, which included: Two intradermal injections of 0.1 mL of Purified Vero Cell Rabies Vaccine (PVRV) on Days 0, 3, 7, and 28. According to WHO guidelines, patients with Category III exposure also got Rabies Immunoglobulin (RIG) on Day 0.Classification of wounds all animal bite cases were classified according to the World Health Organization (WHO) standard for animal bites wounds and PEP indication.

[1] Category I - Touching or feeding animals, licking unbroken skin (no exposure; no PEP needed).

[2] Category II: Nibbling on skin that isn't covered, small scratches or abrasions that don't bleed (minor exposure; vaccination is recommended).

[3] Category III: One or more bites or scratches on the skin, contamination of the mucous membrane or broken skin with saliva (severe exposure;vaccination with RIG recommended)

## Collecting data:

The ARC Register at the Tertiary Care Hospital in Khandwa was used to collect data from the past. We took out all the relevant information and wrote it down on a pre-made data collection form.

We got the following variables from the records:

[1] Demographic information, such as the age and sex of the person who was bitten by an animal.

[2] The date of the first visit (Day 0) and the dates of the follow-up visits (Day 3, Day 7, Day 28).

[3] Type of animal bite wound (Category I, II, or III).

[4] How many doses of the anti-rabies vaccine each patient got

[5] Finishing or not finishing the full ARV schedule.

Variable of Outcome The main goal was to follow the full PEP schedule, which meant getting all four doses of intradermal rabies vaccine on Days 0, 3, 7, and 28. Dropout, or loss to follow-up, was defined as not showing up for any dose after Day 0.

## Analysis of statistics:

We used Microsoft Excel and SPSS version 22.0 to enter and analyse the data. We showed categorical variables as frequencies and percentages. Continuous variables were represented as mean ±standard deviation (SD). The chi-square test was utilized to analyse relationships among categorical variables. A p-value of less than 0.05 was deemed statistically significant. Ethical Consideration of the study was carried out subsequent to receiving authorization from the Institutional Ethics Committee (IEC). Because the study was retrospective and based on records, patient privacy and anonymity were strictly upheld during the data collection process. There was no direct interaction with patients.

## Results and Discussion:

The present study comprised a total of 850 patients with animal bites. Table 1 shows that the age group that was most affected were 21 to 30 years old, with 203 patients (23.9%). The next age group that was most affected was 11 to 20 years old, with 158 patients (18.6%). There were only 5.9% (n=50) of people aged 61 and older (≥61 years). The average age was 29.75 years, and the youngest person was 1 year old and the oldest was 85 years old. These findings are largely consistent with the study conducted by Gogtay *et al.* [15] on tertiary care in Mumbai. They discovered that the majority of individuals bitten by animals were working-age adults, exhibiting a comparable inclination towards younger adults. Sudarshan *et al.* [16] conducted a national metacentric survey in India, indicating that younger and economically active age groups exhibited the highest incidence of animal bites, which they attributed to heightened occupational and recreational outdoor exposure. The elevated incidence in the 21-30-year age group in this study is likely attributable to young adults' increased outdoor activities, engagement in higher-risk occupations, and diminished risk perception. In a retrospective study conducted in Delhi, Jethani *et al.* [17] similarly found that most of the people who went to an animal bite clinic at a tertiary hospital were young people or young adults.

In the present study monthly distribution of animal bite (April to September 2023) (Table 2), the maximum number of animal bite cases was reported in May (17.2%), followed by April (16.7%) and July (16.2%), indicating higher burden during warmer months. Similar seasonal variation was reported by Thahaby *et al.* [18], where animal bite cases peaked during the summer season, particularly in April to May had recorded the highest number of cases (29.3%) followed by autumn season (27.82%). In South Africa, Ishaya *et al.* [19] reported high number of cases in January to March and July to September. This variation can be due to different seasonal pattern and climate condition in African country. In Distribution of cases by WHO category of Animal bite (Table 3) the majority of animal bite cases belonged to WHO category II (84%), followed by Category III (15%), while Category I exposures constituted only 1 % of cases. The predominance of Category II bites may be attributed to increased minor scratches and superficial bites occurring during routine human-animal interactions, prompting healthcare seeking for post-exposure prophylaxis. However, the substantial proportion of Category III bites in the present study highlights the continues risk of severe exposure requiring timely administration of rabies immunoglobulin along with vaccine to prevent fatal rabies infection. Of the 850 patients who initiated ARV under the Updated Thai Red Cross Regimen (four doses), only 381 (44.8%) completed all four doses, yielding an overall dropout rate of 55.2% (n=469) (Table 4). Progressive attrition was evident at each dose interval: 13.4% (n=114) discontinued after Dose 1, 16.8% (n=143) after Dose 2 and 24.8% (n=211) after Dose 3, with the steepest single-point dropout occurring immediately before the final dose. This pattern is particularly concerning because immunological protection under the Thai Red Cross regimen depends on completion of all four scheduled visits.The 55.2% dropout rate in the present study is substantially higher than figures reported elsewhere. Debnath *et al.* [9] in a systematic review and meta-analysis of Indian studies reported a pooled PEP completion rate of 57%, with dose-wise compliance declining progressively across successive visits. Panda *et al.* 20] similarly reported suboptimal compliance to anti-rabies post exposure prophylaxis among animal bite victims, emphasizing that adherence declined with subsequent doses and highlighting poor awareness, logistic barriers, and inadequate follow-up as important contributors to incomplete vaccination. N'Guessan *et al.* [10] similarly found that fewer than half of patients initiating PEP in Côte d'Ivoire completed treatment, identifying perception bias, distance from vaccination centres, and financial constraints as important barriers. In contrast, Shi *et al.* [21] reported a non- completion rate of 26.8% in their cohort, which was attributed to stronger healthcare systems, better patient education, and the availability of animal testing that allowed medically justified early discontinuation in selected situations. In low- and middle-income settings such as the present study, no equivalent safety net exists; Mallewa *et al.* [22] further showed that incomplete or inadequate PEP remained an important feature among laboratory-confirmed rabies deaths in high-burden settings. Female patients demonstrated a higher ARV completion rate (49.3%) than male patients (43.3%), with lower attrition at Dose 1 (9.9% vs. 14.6%) and Dose 2 (16.0% vs. 17.1%) (Table 4).Dropout after Dose 3 was nearly identical across sexes (females 24.8%, males 25.0%), indicating that the major barrier to the final dose may be common to both groups rather than sex-specific. The relatively better adherence among females in the earlier visits may reflect greater perceived seriousness of treatment, better family support for repeat visits, or more consistent health-seeking behaviour. Shi *et al.* [21] observed that females constituted a larger proportion of the incompletely vaccinated cohort in their study, which contrasts with the present findings. This difference likely reflects contextual variation in healthcare access, sociobehavioural patterns, and population characteristics, suggesting that sex-based compliance trends are not uniform across settings and should be interpreted within the local social and health-system environment. Table 5shows that the 21-30-year age group had the highest dropout rate (23.9%), whereas patients aged 61 years and above had the lowest attrition (6.2%). This indicates a particularly vulnerable pattern in young adults, who are both highly exposed and less likely to complete the full vaccination schedule. Such a combination creates a compounded public health risk in the most economically active segment of the population. Debnath *et al.* [9] identified younger age as an important predictor of PEP non-completion in Indian studies, likely because occupational mobility, time constraints, and low perceived risk interfere with follow-up. N'Guessan *et al.* [10] also reported significantly higher odds of PEP discontinuation among patients aged 25-29 years, which is consistent with the peak dropout observed in the 21-30-year group in the present study. The lower dropout among older adults may be related to fewer occupational constraints, stronger family support, and greater concern regarding health consequences. Pardeshi *et al.* [23] have therefore emphasized the need for system-level interventions in India, including reminder systems, digital tracking, and strengthened follow-up mechanisms to improve regimen completion.

## Conclusion:

We found a low ARV completion rate of 44.8% and a high dropout rate of 55.2%, primarily seen in young adult males aged 21-30 years. Most exposures were in Category II (84%) with attrition peaking right before the final ARV dose, indicating low compliance late in the PEP regimen. To help India achieve the WHO’s 2030 goal of zero human rabies death, targeted intervention such as detailed patient counseling at every visit, SMS appointment reminders, and community awareness activity are needed.

## Figures and Tables

**Table 1:** Demographic characteristics of animal bite patients

**Demographic Variable**	**Number of Patients (n = 850)**	**Percentage (%)**
Age Group (Years)		
1-10	136	16.00%
11-20	158	18.60%
21-30	203	23.9% (Maximum)
31-40	137	16.10%
41-50	91	10.70%
51-60	75	8.80%
61 and above	50	5.90%
**Sex**		
Male	637	74.90%
Female	213	25.10%

**Table 2:** Monthly distribution of animal bite cases (April-September 2023)

**Month**	**Number of Patients**	**Percentage (%)**
Apr-23	142	16.70%
May-23	146	17.2%(Maximum)
Jun-23	125	14.70%
Jul-23	138	16.20%
Aug-23	134	15.80%
Sep-23	136	16%
Total	850	100%

**Table 3:** Distribution of Cases by WHO Category of animal bite

**Category**	**Description**	**Number of Patients**	**Percentage (%)**
Category I	Touch/lick on intact skin	~8	~1%
Category II	Minor scratch/nibble on uncovered skin	714	84% (Maximum)
Category III	Transdermal bite/mucous membrane exposure	128	15%
Total		850	100%

**Table 4:** ARV Schedule compliance and dose-wise breakdown by sex (Updated Thai Red Cross Regimen)

**Sex**	**Total Patients**	**Completed ARV (All 4 Doses)**	**Total Incomplete (Dropouts)**	**Dose 1 Only**	**Dose 2 Only**	**Dose 3 Only**
Male	637 (100%)	276 (43.3%)	361 (56.7%)	93 (14.6%)	109 (17.1%)	159
Female	213 (100%)	105 (49.3%)	108 (50.7%)	21 (9.9%)	34(16%)	53 (24.8%)
Total	850 (100%)	381 (44.8%)	469 (55.2%)	114 (13.4%)	143 (16.8%)	212 (24.9%)

**Table 5:** Dropout distribution by age group

**Age Group (Years)**	**Dropout Percentage (%)**
1-10	15.50%
11-20	19.80%
21-30	23.9% (Highest)
31-40	17.40%
41-50	9.90%
51-60	7.30%
61 and above	6.2% (Lowest)
Total Dropouts	465 (54.7%)
